# Reply: Severe Acute Respiratory Failure Associated With Trimethoprim/Sulfamethoxazole Among Adolescent and Young Adults: An Active Comparator-Restricted Disproportionality Analysis From the FDA Adverse Event Reporting System (FAERS) Database

**DOI:** 10.1177/10600280251350681

**Published:** 2025-06-26

**Authors:** Fatemeh Ahmadi, Niaz Chalabianloo, Flory T Muanda

Periyasamy and Mirunalini have offered a pertinent Letter to the Editor regarding our study, “Severe Acute Respiratory Failure Associated With Trimethoprim/Sulfamethoxazole Among Adolescents and Young Adults: An Active Comparator-Restricted Disproportionality Analysis From the FDA Adverse Event Reporting System (FAERS) Database.”^1^ We agree that methodological rigor and transparency are essential in pharmacovigilance research, particularly when interpreting data from spontaneous reporting systems. We appreciate the opportunity to clarify several key aspects of pharmacovigilance, particularly regarding the scientific role of spontaneous adverse event reporting systems, which appear to have been misunderstood in the critique.

First, the OpenFDA initiative is a government-supported platform designed to improve public access to FDA data sets by offering a user-friendly Application Programming Interface (API) and a structured format for data retrieval. Although we accessed the data via OpenFDA for usability, it is important to note that OpenFDA is merely an interface; the underlying source of all reports remains FAERS—the primary post-marketing safety database used by the FDA.^2^

Second, it appears that the core purpose of pharmacovigilance studies using spontaneous reporting systems has been misinterpreted in the comment. Contrary to the assumption that such data sets lack credibility, there is broad consensus in the scientific community that these systems, like FAERS, are indispensable for early signal detection, especially in the context of rare but serious adverse drug reactions (ADRs) with a strong drug-attributable component.^3^ As highlighted by Cutroneo et al, these events often escape detection in clinical trials and structured health care databases, making FAERS the optimal data source for timely risk identification. The volume and accessibility of FAERS data further enhance its value by supporting reproducible signal detection using disproportionality analysis (DA). Several important safety signals have been identified or supported through FAERS data, including associations such as rofecoxib (Vioxx) with cardiovascular events, thalidomide with venous thromboembolism, and pioglitazone with bladder cancer. These examples underscore the essential role of FAERS in pharmacovigilance for detecting and monitoring serious ADRs.^4^

Indeed, DA has been recognized as an essential, hypothesis-generating tool that contributes meaningfully to post-marketing safety surveillance, enabling the identification of unexpected. While not intended to quantify risk or confirm causality in isolation, well-conducted DAs frequently trigger regulatory action, label changes, and further targeted research. The methodological rigor and clinical judgment required to interpret these signals are well documented. Therefore, dismissing the credibility of analyses based on the FAERS risks overlooking an essential aspect of pharmacovigilance infrastructure.^5^

**Figure 1.**
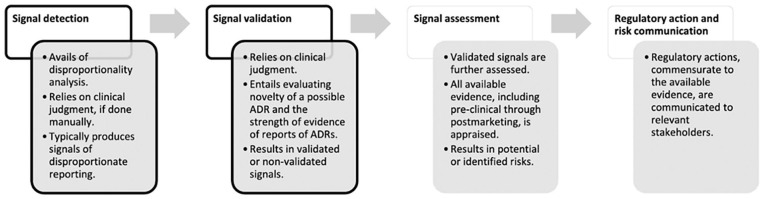
Flowchart of signal management, simplified and applied to reports of suspected ADRs.^5^

To contextualize our study further within the accepted pharmacovigilance framework, we refer to the signal management process outlined by Cutroneo et al. This framework (see Figure 1) identifies “signal detection” as the initial and foundational step in the broader pharmacovigilance workflow. It is at this stage that DA, the method employed in our study, plays a central role. The DA is widely acknowledged as a statistically sound and cost-effective method for screening large spontaneous reporting data sets, such as FAERS, to identify drug-event combinations reported at unexpectedly high frequencies.

Importantly, this signal detection step is never intended to be conclusive or regulatory in isolation. As the figure and accompanying discussion in the article emphasize, multiple critical steps follow detection, including signal validation, clinical review, prioritization, and eventually regulatory decision-making. Each of these stages incorporates additional layers of evidence, expert judgment, and contextual evaluation. The failure to appreciate this multistep process risks conflating early hypothesis generation with regulatory endorsement, a distinction that lies at the heart of sound pharmacovigilance practice.

Therefore, our study should be understood as part of the initial signal detection phase within the pharmacovigilance lifecycle, designed to identify potential safety concerns that warrant further evaluation, not to establish causality or prompt regulatory action. As Cutroneo et al. emphasize, DA “is typically employed in the earliest stage of signal management” and will “give rise to false positives,” underscoring its role as a screening tool, not a verdict. Effect measures in the DA studies, “cannot estimate risks or necessarily account for a causal association” but rather serve to generate hypotheses requiring deeper scrutiny.

Conflating signal detection with causal inference is a misapplication of method and purpose; pharmacovigilance is not intended to replicate the evidentiary standards of randomized trials or epidemiological studies. It functions instead as a frontline surveillance system, capable of detecting rare ADRs under real-world conditions, often missed in pre-marketing assessments. The signals it yields must be interpreted within the broader framework of signal management, with causality assessed only through subsequent validation steps using complementary data sources.

Misinterpreting a pharmacovigilance signal as a causal conclusion not only misrepresents the methodology but also risks undermining the value of the entire post-marketing surveillance framework, which depends on early, non-confirmatory alerts to safeguard public health.

We stand by the scientific merit of our study and reaffirm that, despite its inherent limitations, as acknowledged in our manuscript, spontaneous adverse event reporting remains a cornerstone of post-marketing pharmacovigilance. In summary, FAERS is a credible and essential source for generating safety hypotheses. However, we agree that subsequent confirmatory studies using complementary data sources are necessary to inform regulatory decisions and establish causality.

We welcome ongoing discourse grounded in methodological literacy and thank the editors once again for facilitating this exchange.


Fatemeh Ahmadi, PharmD, MSc *ICES, London, ON, Canada* *Department of Epidemiology and Biostatistics, Western University, London, ON, Canada* *Lawson Health Research Institute, London Health Sciences Centre, London, ON, Canada*
Niaz Chalabianloo PhD *ICES, London, ON, Canada* *Department of Physiology and Pharmacology, Western University, London, ON, Canada.*
Flory T Muanda, MD, PhD *ICES, London, ON, Canada* *Department of Epidemiology and Biostatistics, Western University, London, ON, Canada* *Department of Physiology and Pharmacology, Western University, London, ON, Canada.* *Lawson Health Research Institute, London Health Sciences Centre, London, ON, Canada*

